# Effect of Pulsed Electromagnetic Fields (PEMFs) on Muscular Activation during Cycling: A Single-Blind Controlled Pilot Study

**DOI:** 10.3390/healthcare11060922

**Published:** 2023-03-22

**Authors:** Aurelio Trofè, Alessandro Piras, David Muehsam, Andrea Meoni, Francesco Campa, Stefania Toselli, Milena Raffi

**Affiliations:** 1 Department for Life Quality Studies, University of Bologna, 47921 Rimini, Italy; 2 Department of Biomedical and Neuromotor Sciences, University of Bologna, 40126 Bologna, Italy; 3 Department of Experimental, Diagnostic and Specialty Medicine, University of Bologna, 40126 Bologna, Italy

**Keywords:** PEMF, electromyography, lactic acid, cyclist, performance, physical exercise

## Abstract

Purpose: PEMF stimulation results in a higher O_2_ muscle supply during exercise through increased O_2_ release and uptake. Given the importance of oxygen uptake in sport activity, especially in aerobic disciplines such as cycling, we sought to investigate the influence of PEMF on muscle activity when subjects cycled at an intensity between low and severe. Methods: Twenty semi-professional cyclists performed a constant-load exercise with randomized active (ON) or inactive (OFF) PEMF stimulation. Each subject started the recording session with 1 min of cycling without load (warm-up), followed by an instantaneous increase in power, as the individualized workload (constant-load physical effort). PEMF loops were applied on the vastus medialis and biceps femoris of the right leg. We recorded the electromyographic activity from each muscle and measured blood lactate prior the exercise and during the constant-load physical effort. Results: PEMF stimulation caused a significant increase in muscle activity in the warm-up condition when subjects cycled without load (*p* < 0.001). The blood lactate concentration was higher during PEMF stimulation (*p* < 0.001), a possible consequence of PEMF’s influence on glycolytic metabolism. Conclusion: PEMF stimulation augmented the activity and the metabolism of muscular fibers during the execution of physical exercise. PEMF stimulation could be used to raise the amplitude of muscular responses to physical activity, especially during low-intensity exercise.

## 1. Introduction

Pulsed electromagnetic fields (PEMFs) are a non-invasive medical therapy used for clinical treatments. PEMFs for non-union fracture repair were approved for human use in 1979 by the Food and Drug Administration (FDA). Despite the long time of use, the effects of PEMF are still discussed, as well as the therapeutic benefit for human subjects and further studies are still needed, to confirm the positive influence of a pulsed electromagnetic field [1,2,3]. PEMF therapy is now in use for the treatment of bone conditions such as osteoporosis [4] and fracture [5,6,7]. Due to the piezoelectric effect, PEMFs improve bone mass and density, through the stimulation of osteoblastogenesis with modulation of calcium storage and mineral metabolism. Other studies have shown that PEMFs can improve the tissue oxygenation, microcirculation and angiogenesis in rats, in human erythrocytes and in cell-free assays [8,9]. Such responses could be caused by a modulation of nitric oxide signaling [10] and by the interaction between PEMFs and Ca^2+^/NO/cGMP/PKG signaling [11,12]. In humans, the effects of a pulsed electromagnetic field on blood circulation appear unclear. Rikk at al. [13] showed that PEMF treatment reduced the systolic blood pressure in aging adults, but not the diastolic pressure or arterial stiffness, suggesting that PEMFs could influence the peripheral resistance and microcirculation. Kwan et al. [14] found PEMF therapy helpful in patients with diabetes, due to the increased microcirculation by enhancing the capillary blood velocity and diameter. Sun et al. [15] showed that PEMFs improved the blood flow velocity of the smallest veins without changing their diameter. Nevertheless, further investigation is needed for an accurate description of the interactions between pulsed electromagnetic fields and human cells and tissue and to better understand the effects of the stimulation parameters such as time and frequency. It has been hypothesized that the different responses to PEMF therapy depends on the biological tissue or dosage of stimulation of a specific electromagnetic signal [16].

Despite much research and several medical applications, few studies have investigated the effects of PEMFs during physical activity. Galace de Freitas et al. [17] suggested that the combination of exercise training and PEMF stimulation could be used to improve the function, muscle strength and decrease in pain of patients with shoulder impingement syndrome. However, the benefits deriving from the association of PEMF and training are still controversial and more evidence is necessary to confirm the positive influence of pulsed electromagnetic fields.

Parhampour et al. [18] applied PEMFs in association with six weeks of a resistance training program in patients with severe hemophilia A and osteoporosis in order to improve their muscle strength, bone formation and joint function. The results showed that PEMF stimulation, in association with resistance training, could be more efficient than PEMF therapy alone in improving bone formation due to the increased level of serum bone-specific alkaline phosphatase. However, further investigation is needed to clarify the benefits deriving from the association of PEMFs and training.

Grote et al. [19] investigated short-term PEMF stimulation on heart rate variability in the recovery phase after physical exercise, suggesting a possible influence on the autonomic system. In this study, twenty minutes of exposure to low-frequency PEMFs accelerated the recovery of heart rate variability, especially in the very-low-frequency range, with a more rapid return to the initial sympathetic tone. Despite that, the basal autonomic tone seems to play a crucial role as well as the power of the electromagnetic signal. Further studies are necessary to determine the influence of pulsed electromagnetic fields on the autonomic system and recovery.

Jeon et al. [20] investigated the effects of PEMF therapy on pain, soreness or muscle force generation associated with delayed-onset muscle soreness (DOMS) during recovery after isometric exercise. PEMF treatment on the brachii biceps for ten minutes after training reduced the severity of perceived symptoms of DOMS in the following days, enhancing the quality of recovery. PEMF treatment also increased the median frequency of muscle activation and reduced the electromechanical delay during isometric contraction in the day after exercise, suggesting a shortened recovery time. Despite this, no effect was found on the peak of isometric force generation; thus, more studies are necessary to confirm the positive influence of PEMF to improve and accelerate the recovery phase.

Furthermore, the effect of PEMF therapy for pain in the shoulder [21] and neck [22] require additional studies in order to better clarify the benefit of stimulation.

The aim of this study was to help better clarify the influence of PEMFs in humans and investigate the effect during physical activity. Until now, very few studies have investigated the influence of PEMF stimulation during exercise or sport activity. Given the importance of oxygen uptake in sport activity [23], especially in aerobic disciplines (e.g., cycling), we sought to investigate the PEMF’s effect during exercise to assess its influence on muscular activity. We know from our previous study that PEMF stimulation results in a higher O_2_ muscle supply during exercise through increased O_2_ release and uptake [24]. We thus hypothesize that PEMF stimulation could improve the muscular response due to a higher amplitude of muscular activity generated by the enhancement of muscular contraction mechanisms.

## 2. Materials and Methods

### 2.1. Subjects and Design

The study design was a single-blind, randomized controlled trial. The experiments were performed in 20 male semi-professional cyclists (mean ± SD: age 22.3 ± 5.7 years; body mass index 22.5 ± 2.7; VO_2 max_ 54.7 ± 10.4 mL/min/kg; weight 71.5 ± 10.3 kg; height 178.1 ± 6.5 cm). All subjects were volunteers, healthy, non-smokers and none of them were taking medications or supplements. None of the subjects reported a physical deficit or muscular injury at the time of the study. All participants received a verbal explanation of the experimental procedures, and informed consent was obtained before the beginning of recordings. The experimental protocol was approved by the Institutional Bioethic Committee of the University of Bologna. The experiments were performed in accordance with the ethical standards laid down in the 1964 Declaration of Helsinki. Table 1 shows the features of each participant of the study.

### 2.2. Methodology

For the realization of this study, we recorded the electromyography (EMG) activity from the vastus medialis (RVM) and biceps femoris caput longum (RBF) of the right leg. The EMG data were recorded with a data sampling rate of 1000 Hz with a Free-EMG 1000 (BTS Bioengineering, Inc.). Electrodes were placed on the muscular belly of each muscle. To improve contact, the skin was shaved and cleaned with ethanol before placing the Ag/AgC1 disposable electrodes 32 × 32 mm with active area of 0.8 cm^2^ and inter-electrode distance of 2 cm used in bipolar configuration (RAM, s.r.l, Italy). The athletes visited our laboratory three times, with three days between each visit, in which we performed different recordings. We first recorded the maximum voluntary contraction (MVC) used to normalize the electromyographic data. We normalized the EMG activity to the peak of the MVC following the same protocol used in previous studies [25,26]. Then, through an incremental test on a cycle-ergometer (H-300-R Lode), we determined the ventilatory threshold (VT), maximal oxygen consumption (VO_2 max_) and individualized workload for the succeeding recording sessions. The protocol for the incremental test was the following: each subject cycled at 50 Watt for 5 min, followed by a workload at 80 watt that increased by 20 Watt every 1 min, at a cadence of 70 RPM, until the volitional exhaustion [27]. Expired gas was analyzed using a Quark b2 breath-by-breath metabolic system (Cosmed srl, Rome, Italy). The individualized workload for each athlete (mean ± SD: 307.1 ± 60.1 watt) corresponded to ~50% of the difference between the power (watt) reached at ventilatory threshold (VT) and at VO_2 max_ (~50% Δ VT−VO_2 max_) in order to provide a heavy intensity of exercise [28].

After the initial session, the subjects came to the laboratory for two more sessions on separate days, in which they performed a constant-load exercise with randomized active (ON) or inactive (OFF) PEMF stimulation. In order to stimulate the entire thigh, two PEMF loop-antenna devices (Torino II, Rio Grande Neurosciences, USA) were positioned on the right leg at the beginning and at the ending of the thigh. The subjects were blinded to the ON/OFF stimulations (single-blind trial). The PEMF waveform consisted of a pulse-burst modulated 27.12 MHz sinusoidal carrier, with 2 ms burst width repeated at 2 HZ, with a peak magnetic field at the center of the loop 5 ± 1 µT. The measurements were performed on the same cycle-ergometer in a quiet room with a stable and comfortable temperature (22 °C), at the same time of the day (9:00–12:00 AM) to avoid circadian influence. The subjects were instructed to avoid strenuous activity and alcohol in the 24 h preceding the test. The athletes were asked to avoid drinking caffeinated beverages before the experimental procedures. Each subject started the recording session with 1 min cycling without load (0 Watt), which was called the phase of warm-up, followed by an instantaneous increase in power, which was called the phase of constant-load physical effort. Each trial was ended intentionally, as time to exhaustion, when athletes were unable to keep the constant-load physical effort.

We also measured the blood lactate concentration before the beginning of each trial (lactate baseline) and at the third minute of the constant-load exercise.

### 2.3. Data Analysis

**Figure 1:**
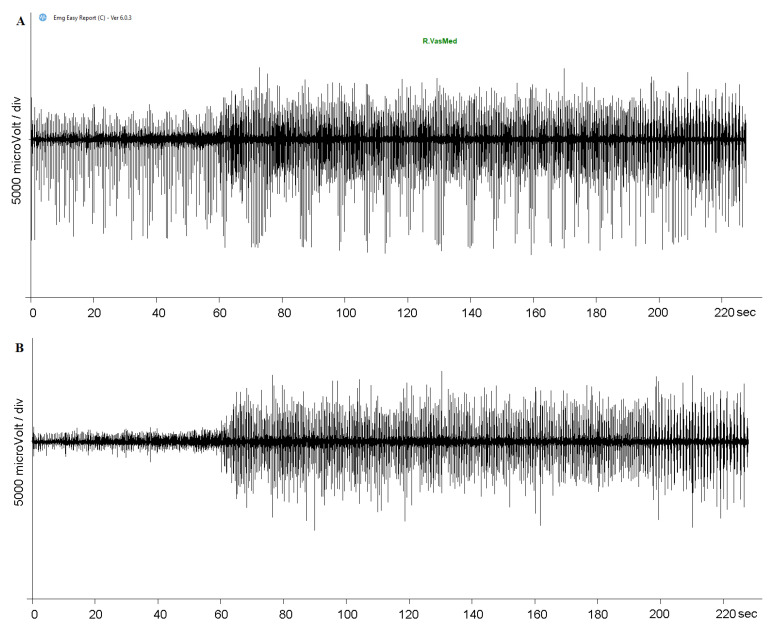
PEMF artifact removal with Easy Report 6.03.8 (Merlo Bioengineering, Italy). The raw EMG trace (**A**) was processed using a wavelet-based denoising filter and a specific algorithm in order to return a trace without PEMF artifacts (**B**).

**Figure 2:**
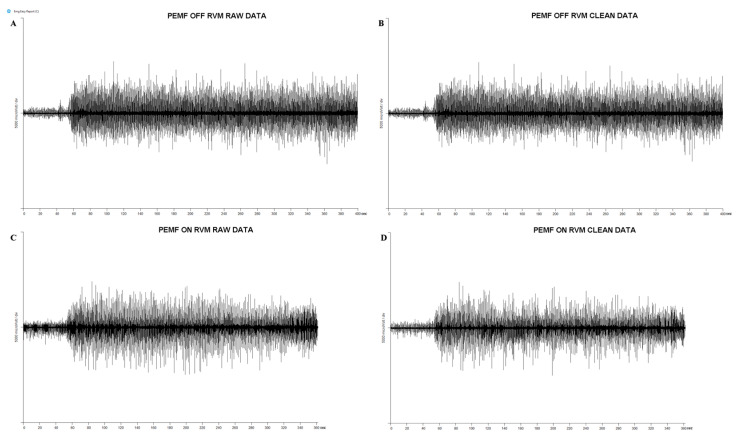
Raw and clean EMG traces for a typical subject in both OFF/ON PEMF stimulation. (**A**) Raw data for a typical muscle (right vastus medialis) during PEMF OFF stimulation. (**B**) Clean data for a typical muscle (right vastus medialis) during PEMF OFF stimulation: we used a wavelet-based de-noising filter in order to reduce background noise and automatically remove large and frequent artifacts on EMG traces. (**C**) Raw data for a typical muscle (right vastus medialis) during PEMF ON stimulation. (**D**) Clean data for a typical muscle (right vastus medialis) during PEMF ON stimulation: after wavelet-based de-noising filter, we used a consolidation process and a specific algorithm in order to remove PEMF artifacts.

The software EMG easy report 6.03.8 (Merlo Bioengineering, Italy) was used for EMG traces on data process and artifact removal [29,30,31]. First, we used a wavelet-based denoising filter, in order to reduce the background noise and automatically remove large and frequent artifacts [32]. After detecting and removing specific PEMF artifacts on the EMG traces, a consolidation process described below was applied [32,33,34]. Starting from the raw signal, a peak emphasis operator, called Smoothed Non-Linear Energy Operator (SNEO) [33], was applied. SNEO is similar to the Taeger–Kaiser, the other operator frequently used with EMG signals [34]. The peak positions and amplitudes were found using thresholds of the minimum amplitude and distance between PEMF peaks. The position of unrecognized stimulus was found with linear interpolation of the values obtained in the previous point. When the artifact positions were found, the parts of the signal 20 ms before and 80 ms after the peaks were forced to zero (Figure 1). After that, the algorithm calculated the amplitude of the RMS limited at the signal of the muscle activity for each detected onset interval. The activation intervals were calculated through a specific algorithm [32] using a mean background noise level of 10 uV RMS. Then, the values were normalized to the peak of the MVC (Figure 1). For all the variables recorded, we averaged the values of all subjects in the PEMF ON and PEMF OFF stimulation. Figure 2 shows the raw and clean EMG traces for a typical subject, recorded during warm-up and during physical effort, for a typical investigated muscle (RVM), in both OFF/ON PEMF stimulation.

We compared the mean values of muscular activity for unloaded cycling (warm-up), constant-load exercise (constant-load physical effort) and muscular activation related to the exercise duration (activity/time to exhaustion). A 2 (muscles, RVM and RBF) × 2 (stimulation, PEMF ON and PEMF OFF) repeated-measures ANOVA was performed on each condition (warm-up, constant-load physical effort, activity/time to exhaustion) separately. The effect sizes were calculated using partial eta squared (η2p), and the means were considered significantly different at *p* < 0.05.

The blood lactate levels were analyzed with Student’s t-test for paired data with the means considered significantly different at *p* < 0.05. The effect sizes (ES) were calculated as the mean difference standardized by the between-subject standard deviation and interpreted according to the following thresholds: <0.20; small, >0.20–0.60; moderate, >0.60–1.20; large, >1.20–2.00; very large, >2.00–4.00; extremely large, >4.00 [35]. Data were analyzed with SPSS v22.0 (IBM, New York, NY, USA).

## 3. Results

**Figure 3:**
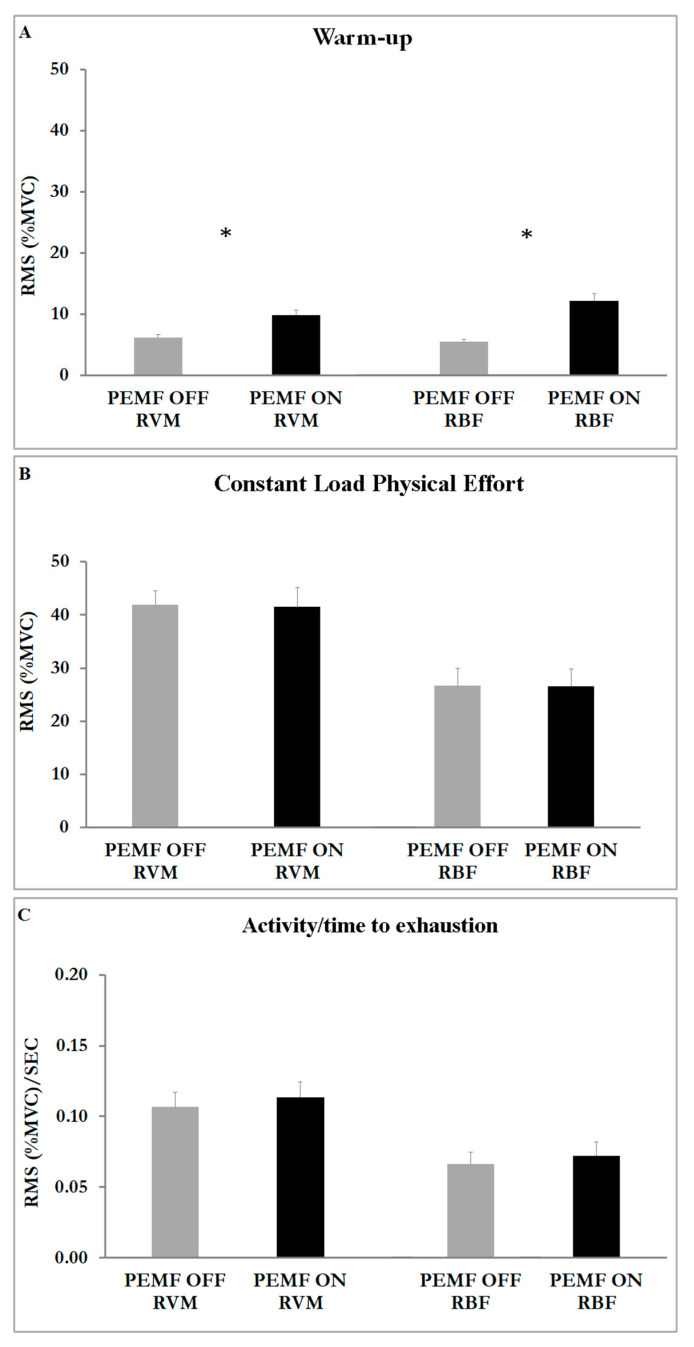
Histograms represent the root mean square (RMS) of the normalized EMG values (mean ± SEM) of both muscles (RVM; RBF) in both stimulations (ON; OFF). (**A**) EMG activity at warm-up. (**B**) EMG activity at phase of constant-load physical effort. (**C**) EMG activity related to the time of exhaustion. Asterisks indicate significant differences at *p* < 0.05.

Figure 3 shows the effect of PEMF stimulation. We compared PEMF ON and PEMF OFF within the same muscle and the results showed significant differences: PEMF ON exhibited a higher significant RMS value of both muscles with respect to PEMF OFF during warm-up (RVM t(16) = −5.61; *p* < 0.001; ES 0.57—moderate; RBF t(16) = −6.29; *p* < 0.001; ES 0.69—large) (Student t-test: RVM *p* < 0.001; RBF *p* < 0.001) (Figure 3A). We also found a greater amplitude on RVM in comparison to RBF in both stimulations during warm-up (Figure 3A), during constant-load physical effort (Figure 3B) and in the relationship between muscular activity and exercise duration (Figure 3C).

The ANOVA results showed a significant main effect for muscle (F1,17 = 16.452; *p* < 0.001; η2p = 0.141) and condition (F1,2 = 381.942; *p* < 0.001; η2p = 0.884). The analysis also showed an interaction effect of muscle x condition (F2,16 = 20.133; *p* < 0.001; η2p = 0.287).

We compared the mean duration of each trial (time to exhaustion) in both ON/OFF PEMF stimulation and we did not find any significant difference (ON = 383 ± 15 s; OFF = 413 ± 16 s) in a one-way ANOVA with PEMF (ON/OFF) as a factor (F1,1 = 1.939; *p* = 0.171807).

**Figure 4:**
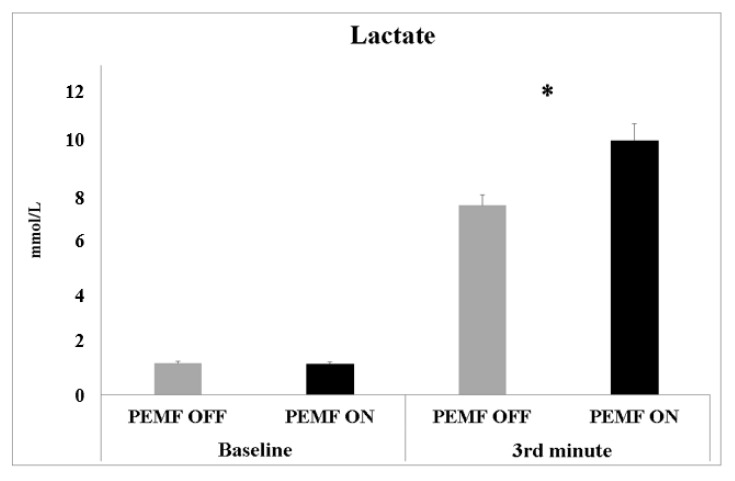
Blood lactate concentrations measured before the beginning of the experiment (baseline) and at 3rd min of constant-load physical effort of both PEMF ON and OFF stimulation. Data are shown as mean values ± SE. Asterisks indicate significant values at *p* < 0.05.

We measured the blood lactate concentration (mmol/L) before the beginning of the exercise session (baseline) and at the third minute of the constant-load physical effort (Figure 4). The analysis showed a significant difference between PEMF ON (10.05 ± 0.65 mmol/L) and PEMF OFF (7.48 ± 0.42 mmol/L) for the lactate concentration recorded during the constant-load exercise (t(19) = −4.78; *p* < 0.001; ES 0.46—moderate).

## 4. Discussion

The main result of this study is that PEMF stimulation increases the activity of muscle fibers during warm-up but not during high-intensity constant load.

### 4.1. Effect of PEMF Stimulation on Warm-Up (Low Intensity)

During warm-up, when athletes cycled at a very light aerobic intensity, PEMF stimulation enhanced the activity of both the vastus medialis and biceps femoris (Figure 3A). One possible explanation for this effect arises from the change in the membrane permeability and Ca^2+^ channel conduction enhancing the ion flux and cellular concentration [36,37]. The increase in the amplitude of the muscular response was probably caused by the effect of stimulation on type-I and type-II muscular fibers. Likely, PEMF stimulation increased the activity of type-II fibers, normally poorly activated during light physical effort, suggesting a possible application of PEMFs during the preparatory phase before competition, in order to raise the magnitude of muscular response.

During the constant-load phase of effort, PEMF stimulation did not affect the amplitude of muscle activity (RMS). The analysis showed significantly increased activity for the vastus medialis with respect to the biceps femoris (Figure 3B). This result is not surprising given that the effective role of the vastus medialis during cycling is well-known, but the role of the biceps femoris is still under discussion: the magnitude of the biceps femoris is more affected by fatigue, pedaling rate, coordination/activation timing (angle), training status, shoe–pedal interface and body position. The biceps femoris is a bi-articular muscle involved in knee flexion and hip extension. According to Hug and Dorel, the biceps femoris seems to be more important for energy transfer between joints during cycling rather than to supply the main force [38]. One of the largest activities and an earliest activation of biceps femoris seem to be related to increased fatigue in both the vastus lateralis and medialis as a consequence of modified coordination and activation patterns [38]. In the present study protocol, the workload was instantaneous and strongly near to maximal, causing an immediate and large response of the main muscles of cycling, such as the vastus medialis, causing a rapid increment of its muscular activity. Thus, the biceps femoris increased its activity later, upon the arrival of fatigue in the vastus medialis.

PEMF stimulation has an effect on muscle activity during low-intensity exercises but does not seem to affect muscle during heavy-load exercises. Possibly, the higher muscle activation covered the effect of stimulation. This is reasonable because our subjects performed a strenuous exercise that required a very high muscle activity. Our dosage of stimulation may not have been sufficient to increase even over the amplitude of muscle activity during exercise. Despite this, the higher blood lactate concentration recorded on exercise during stimulation indicates an effect of PEMF on muscle activity, especially on the contraction mechanism and glycolytic metabolism of type-II muscular fibers strongly involved during exercise.

### 4.2. Effect of PEMF Stimulation on Lactate Concentration

The results showed that the PEMF stimulation caused an increase in the blood lactate, suggesting a potential mechanism of microstimulation in enhancing the activity of type-II muscular fibers, typically recruited when the intensity of exercise exceeds the ventilatory threshold. Moreover, lactate production is essential to delay muscle fatigue during heavy physical exercise. According to Robergs et al. [39], lactate production delayed metabolic acidosis and muscle fatigue, preventing the impairment of exercise performance. Lactate prevents pyruvate accumulation and supplies muscles’ production of NAD+, based on ATP regeneration from glycolysis [39]. The high-intensity exercise used in this study, with high increases in power during physical effort, led to a faster reduction in the intramuscular pH, suggesting that PEMF stimulation promoted type-II fiber metabolism and lactate production to delay metabolic acidosis. These results suggest a possible application of stimulation during exercise to enhance the amplitude of muscular fibers in response to physical activity. The results of the present study clearly show an effect of PEMF stimulation on low exercise intensity and on the amplitude of muscular responses (Figure 3A). The higher magnitude of muscular activity seems to suggest that PEMF stimulation could enhance muscular activation during preparatory activity.

In addition to contraction mechanisms, it is possible to hypothesize that PEMF stimulation affected the energetic system inside the muscular fiber, especially glucose utilization. In rats with streptozotocin-induced diabetic muscle atrophy [40], chronic PEMF treatment affected metabolic enzymes in the quadriceps, with increased succinate dehydrogenase (SDH) and malate dehydrogenase activity (MDH), thus suggesting an increase in the metabolic capacity of muscle. Further, it has been found that PEMF treatment reduced blood glucose and increased serum insulin levels. In insulinoma cells, exposure to PEMF attenuated insulin secretion, suggesting effects on the calcium channels and ion flux [41]. In the present study, the high values of lactate recorded during PEMF stimulation were probably due to the increased overall activity of type-II fibers and boosting of their glycolytic metabolism.

### 4.3. Practical Applications

The results of this study show that PEMFs can have an effect on muscular activity, suggesting potential applications in sport disciplines. Based on the present results, PEMF stimulation could be used during light physical effort in order to enhance the amplitude of muscular responses to exercise.

PEMFs might be used at a high intensity of physical effort or during hard work-out sessions in order to boost the glycolytic metabolism of type-II fibers in response to heavy workloads and increase the benefits of an exercise program such as peripheral heart action training [42].

PEMF stimulation could also be applied during warm-up to raise the amplitude of muscular responses during the preparatory activity of different performances such as jumps, shots or sprints. PEMF stimulation could also be applied during light exercise or low aerobic intensity in order to increase the overall muscular response. Finally, due to the effect of PEMFs on succinate and malate dehydrogenase in rat quadriceps, it could be a possible effect of microstimulation on the aerobic activity over short and long distances.

### 4.4. Limitation of the Lactate Measure

The main critical issue of this study regards the lactate measurement. In the methodological preparation of this study, we chose to take the sample before the beginning of the exercise session (baseline) and at the third minute of the constant-load exercise. We chose this moment because it represents the common time of the end of VO_2_ kinetics phase II and the start of the slow component [43,44] in order to obtain a more standardized value compared to that of the end of the exercise, given that the time of exercise differed for each athlete. The results showed a strong PEMF effect on the lactate concentration. The authors are aware that taking more lactate samples during the entire phase of both exercise and recovery, building the entire lactate curve, would better clarify the influence of PEMF on the glycolytic metabolism of type-II muscular fibers during exercise. Future experiments should be aimed to measure lactate every minute during the entire phase of exercise and recovery to better clarify the influence of PEMF on the energetic system. This would allow us to uncover the effects of PEMF stimulation on the glycolytic metabolism of type-II muscular fibers during exercise.

## 5. Conclusions

To the best of our knowledge, this is the first study to investigate PEMF stimulation during exercise performed between low and severe intensity. This study shows an influence of PEMF stimulation on muscle activity, as well as on the energetic system during exercise. Despite this, more studies are necessary to confirm the influence of pulsed electromagnetic fields in human subjects during physical activity. We believe that these first observations could open new horizons in the field of sport performance. Further studies are necessary to elucidate the stimulation parameters necessary to elicit the most useful physiological response.

## Figures and Tables

**Table 1:** Features of each participant of the study.

Athletes	Age (Years)	VO_2 max_ (mL/min/kg)	Workload (Watt)	Weight (kg)	Height (cm)	BMI
A.D.R.	24	56.1	304	69.0	181	21.1
A.M.	15	55.3	310	68.0	182	20.5
D.P.	19	63.7	320	58.5	171	20.0
D.S.	20	47.5	230	78.0	185	22.8
F.D.	27	54.8	286	64.5	169	22.6
G.F.	20	66.7	500	76.0	189	21.4
G.G.	34	36.9	281	89.0	180	27.5
G.P.	27	49.6	285	74.0	175	24.2
I.V.	19	62.0	352	70.0	180	21.6
J.B.	37	36.1	255	88.0	187	25.2
L.P.	16	54.0	285	65.0	166	23.5
L.T.	20	64.4	340	70.0	183	20.9
M.B.	25	37.1	230	81.0	178	25.6
M.G.	18	69.5	340	58.0	183	17.3
M.L.	22	60.5	352	76.0	174	25.1
N.M.	19	66.6	362	64.0	180	19.8
P.L.	23	55.5	240	54.0	168	19.1
R.B.	23	51.9	295	73.5	181	22.4
S.B.	16	63.1	285	63.0	169	22.1
S.C.	21	42.4	290	90.0	180	27.8
Mean	22.3	54.7	307.1	71.5	178.1	22.5
SD	5.7	10.4	60.1	10.3	6.5	2.7
SEM	1.3	2.3	13.4	2.3	1.5	0.6

## Data Availability

The data presented in this study are available on request from the corresponding author. The data are not publicly available due to privacy restrictions.
